# Body image satisfaction and weight concerns among a Mediterranean adult population

**DOI:** 10.1186/s12889-016-3919-7

**Published:** 2017-01-06

**Authors:** Maria del Mar Bibiloni, Josep Ll Coll, Jordi Pich, Antoni Pons, Josep A. Tur

**Affiliations:** Research Group on Community Nutrition and Oxidative Stress, University of Balearic Islands, and CIBEROBN (Physiopathology of Obesity and Nutrition CB12/03/30038), Guillem Colom Bldg, Campus, E-07122 Palma de Mallorca, Spain

**Keywords:** Balearic Islands, Body image, Weight concern, Physical activity, Obesity

## Abstract

**Background:**

People tend to underestimate their current weight and overestimate their height minimizing health risk factors. The aim of this study was to assess body weight satisfaction, acceptance of body image, weight concern and dieting habits among a Mediterranean adult population.

**Methods:**

Cross-sectional survey was carried out (2009–2010) in the Balearic Islands, Spain. A random sample (*n* = 1081) of young (18–35 y.o.) and middle-aged adults (36–55 y.o.) was interviewed and anthropometrically measured.

**Results:**

Women were more dissatisfied being overweight, less dissatisfied being underweight, and more worried about weight gain than men. Middle-aged participants were more dissatisfied with body shape and underestimated body weight than young’s. Employed women defined better current weight than unemployed, but unemployed were more worried about body weight gain. Overweight adults were more likely to underestimate their body weight but were also very worried about weight gain and more likely to report current dieting than their leaner counterparts. Active participants self-reported better body weight and were more satisfied with body image than sedentary.

**Conclusions:**

Most of studied population reported body image dissatisfaction, but half of them are not worried about it. Women were more concerned about their body weight status. Practice of physical activity is a positive factor in self-perception. Holistic strategies are needed to avoid promoting unreal bodies, as well as the acceptance of the real body image.

## Background

Overweight and obesity negatively impact quality of life, increase risk of developing chronic diseases and conditions, and are costly to healthcare systems [1–3]. The prevalence of obesity has tripled in many countries in the World Health Organization (WHO) European Region since the 1980s, and the number of those affected continues to rise at an alarming rate [4]. The prevalence of overweight and obesity in Spain in 2009 was 34.2 and 13.6%, respectively [5], making it one of the European countries with the highest prevalence. Although overweight and obesity has remained mostly stable over the last decade in the Balearic Islands, approximately one in four adults were reported as being overweight while one in ten was obese.

Excessive weight has been associated with a decrease quality of life [6, 7], an increase in serious chronic diseases (hypertension, diabetes, hypercholesterolemia, asthma, among others) [8, 9] and predisposition to some infections [10]. However, weight loss can decrease obesity-related risks; just losing 5–10% of body weight gives health benefits, as lower blood pressure, LDL cholesterol, and triglycerides, among other cardiovascular risk factors [2, 11–13].

In weight control practices, any factor of body self-perception should be considered. An essential determinant of attempts to lose weight is awareness of having excessive weight status, which has a higher impact than the objective current weight status [14]. Earlier studies found that people tended to underestimate their current weight and overestimate their height [15], and artificially lower their risk factor profile which may have negative health outcome implications. Weight, height and body image self-perception may be influenced by many factors: physical, interpersonal, emotional, cultural [16] and socioeconomic, including the mass media (magazines and TV) that increase body image dissatisfaction and then induce subsequent eating disorders [17–19]. The ‘ideal body’ that is often portrayed in popular culture and mass media is a tall and thin frame which typically does not reflect the build of many women in the average population developing sterner judgment of their self-image and sometimes becoming unnecessarily worried about their weight [20–22]. At the same time, the image that the mass media uses of severe obesity sends a confused message that high weights are required in order to define overweight [23]. Better knowledge of the factors of self-perception of body characteristic may be an important factor in weight control practices.

The aim of this study was to assess body weight satisfaction, acceptance of body image, weight concerns and dieting habits among a Mediterranean adult population.

## Methods

### Study design

The study was a population-based cross-sectional nutritional survey carried out in 2009–2010 in the Balearic Islands, Spain, a Mediterranean region. Obesity and Oxidative Stress (OBEX) survey (2009–2010), which was designed to obtain information on the health and nutritional status of the resident population in the Balearic Islands [24]. The target population consisted of all inhabitants of the Balearic Islands aged between 16 and 65 years old. The population sample was taken from residents registered on the Balearic Islands’ official population census. The sampling technique included stratification according to geographical area and municipality size, inhabitants’ age (3 strata) and gender and randomisation into subgroups, with Balearic Islands municipalities being the primary sampling units and individuals within these municipalities comprising the final sample units.

### Selection of participants, recruitment and approval

This analysis was limited to young (18–35 years old) and middle-aged (36–55 years old) adult population (*n* = 1081) that was interviewed one:one. The participation rate was 92.5% during 2009–2010. Non-participation rates included potential participants who declined to be interviewed (particularly over-55’s) as well as involuntary non-participants who were excluded due to unavoidable constraints on them taking part. This sample size was considered sufficient to detect an overweight and obesity prevalence of 40% with 95% confidence level and a precision rate of 3.0%. This sample size was also sufficiently large to estimate the prevalence of 95% confidence and a precision rate of 3.7 and 5.0% in the young (*n* = 704) and middle-aged (*n* = 377) population, respectively. Moreover, this sample size was considered sufficient to detect risk factors with a precision rate of 4.5% in men (young adults: 5.3%; middle-aged adults: 8.5%) and 3.8% in women (young adults: 4.9%; middle-aged adults: 6.0%). Pregnant women were not considered in this study. Characteristics of the participants are shown in Table 1.

**Table 1:** Characteristics of the participants

	All	18–35 y.o.	36–55 y.o.
*n*	1081	704	377
Weight (kg)^a^	64.2 ± 11.9	62.4 ± 12.4	66.9 ± 13.3
Height (cm)^a^	161.6 ± 6.4	162.1 ± 6.4	160.9 ± 6.5
BMI (kg/m^2^)^a^	24.6 ± 4.9	25.9 ± 5.2	23.7 ± 4.4
Prevalence of overweight (%)^b^	24.8	20.9	30.6
Prevalence of obesity (%)^b^	11.0	7.7	15.9
Prevalence of excessive weight^1^ (%)^b^	35.7	28.6	46.4
Origin (%)^b^			
Balearic Islands	66.0	66.4	65.2
Regions of Spain	21.1	18.4	26.1
South America	9.0	11.0	5.4
Other countries	3.9	4.3	3.3
Educational level (%)^b^			
Low	7.2	5.1	11.2
Medium	53.0	58.1	43.6
High	39.8	36.8	45.2
Professional profile (%)^b^			
Student	22.9	34.4	1.6
Unemployed	7.7	6.5	9.9
Employed	69.4	59.1	88.5
Leisure-time physical activity			
Yes	59.1	58.6	60.0
No	40.9	41.4	40.0

*Abbreviations: BMI* body mass index, *NS* not significant

Prevalence of overweight: BMI ≥25- < 30 kg/m^2^; obesity: ≥30 kg/m^2^; excessive weight: BMI ≥ 25 kg/m^2^

Data were expressed as ^a^mean ± standard deviation and ^b^prevalence (%)

### Anthropometric measurements

Anthropometric measurements were performed by two observers who underwent identical and rigorous training as an effort to minimize the effects of inter-observer variation. Data collection took place between September 1, 2009 and June 30, 2010. Height was measured using a mobile anthropometer (Kawe 44444, Asperg, Germany) to the nearest millimetre, with the subject’s head in the Frankfurt plane. Body weight was determined to the nearest 100 g using a digital scale (Tefal, sc9210, Rumilly, France). The participants were weighed in bare feet and light clothes, noting and subtracting the weight of the clothes. Weight and height measures were used to calculate body mass index (BMI, kg/m^2^). Normal-weight, overweight, obesity and excessive weight were defined as BMI 18.5–24.9 kg/m^2^, BMI 25.0–29.9 kg/m^2^, BMI ≥30 kg/m^2^ and BMI ≥25 kg/m^2^ respectively [4].

### Body weight estimation

Body weight estimation was obtained using the difference between self-reported weight minus measured weight. Body weight estimation was defined as “correct” if the absolute difference between self-reported minus measured weight was less than 2.0 kg, as “underestimated” if the difference was lower −2.0 kg and as “overestimated” if the difference was higher 2.0 kg [25].

### Body image perception

**Fig. 1:**
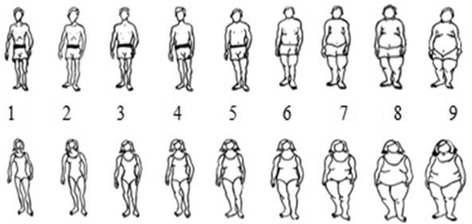
Stunkard scale to measure perceived body image (adapted from [26])

Perceived body image was measured using the Stunkard scale [26], which consists of silhouette drawings ranging from 1 to 9 with monotonic increments in the overweight percentage where 1 is the leanest silhouette and 9 is the largest silhouette. Separate figures for men and women were used (Fig. 1). From the 9 body figures, participants were asked to identify: (a) ‘Which silhouette looks most like yourself?’ and (b) ‘Which silhouette would you like to look like?’ The difference between perceived body image and desired body image was used to determine the level of dissatisfaction with current body image. Values other than zero represent dissatisfaction with perceived body image. A positive value was indicative of the participant’s wish to be thinner than their perceived current size, while a negative value reflected the participant’s wish to be heavier than their current perceived size.

### Weight concern

Weight concern was evaluated using the following question: “Are you worried about weight gain?” and the answers were limited to: ‘Not at all’, ‘A little bit’ and ‘A lot’.

### Current dieting

Current dieting was evaluated using the following question: “Are you currently dieting?”. The answers were limited to: ‘Yes’ or ‘No’.

### Socio-economic and lifestyle factors

A questionnaire including the following questions was used: age group (young adults, aged 18–35; middle-aged adults, aged 36–55); place of birth (Balearic Islands; other regions in Spain; South America; other countries); educational level (grouped according to years and type of education: low, <6 years at school; medium, 6–12 years of education; high, >12 years of education); professional profile (student; unemployed -including homemaker, incapacity, retired-; employed); and leisure-time physical activity (LTPA) was evaluated according to guidelines for data processing and analysis of the International Physical Activity Questionnaire 2.0 [27] in the short form. On the basis of their total weekly (wk) time of moderate or vigorous physical activity, the subjects were divided into 2 groups: no, <150 min/week, and yes, ≥150 min/week.

### Statistics

Analyses were performed with the SPSS statistical software package version 21.0 (SPSS Inc., Chicago, IL, USA). Significant differences were estimated between categories of each of variables listed: Weight Estimation, Weight Concern, Body Image Satisfaction and Current Dieting groups were calculated by chi-square (*χ* ^2^). Significant differences between males and females were also tested by *χ* ^2^. Logistic regression models were used to estimate the odds ratios (OR) and 95% confidence intervals (CIs) to examine the possible associations between body image satisfaction, weight concern and current dieting (dependent variables) and sex, professional profile, BMI distribution and LTPA (independent variables). Statistical significance was considered at an alpha of 0.05.

## Results

### Weight estimation

Around 14% of the study population did not report their current weight (12.9% males; 15.4% females) (Table 2). For both male and female participants, the prevalence of middle-aged adults who underestimated their current weight was higher than youngers, and the prevalence of middle-aged participants who overestimated current weight was lower. Differences in weight estimation by professional profile were also observed in both sexes. Females who were employed were also more likely to report their current weight correctly compared to their unemployed counterparts. For BMI status: 24.9% of overweight/obese males and 40.7% of females did not report their current weight. Underestimation of body weight was observed in 30.8% of excessive weight sample. Underestimation was also shown by around a quarter of overweight males and females, and 45.3% of obese males and 36.8% females. More active males and females reported their current weight correctly.

**Table 2:** Weight Estimation according to socio-demographic variables, BMI distribution and LTPA^a,b,c^

	Underestimation	Correct	Overestimation	Don’t’ know/Don’t answer	*P*
*Men (n = 451)*	20.6	60.5	6.0	12.9	
Age (years)					
18–35	18.1	60.4	7.4	14.1	0.032
36–55	27.2	60.8	2.4	9.6	
Origin					
Balearic Islands	19.8	61.4	5.9	12.9	0.218
Regions of Spain	24.7	61.8	7.9	5.6	
South America	18.5	48.1	7.4	12.7	
Other countries	22.2	55.6	0.0	22.2	
Educational level					
Low	20.7	51.7	13.8	13.8	0.296
Medium	18.9	59.8	6.3	15.0	
High	23.2	62.8	4.3	9.8	
Professional profile					
Student	13.2	52.1	9.9	24.8	<0.001
Unemployed	26.9	57.7	3.8	11.5	
Employed	23.3	64.5	4.4	7.8	
BMI (kg/m^2^)					
Normal-weight	9.9	69.8	7.3	12.9	<0.001
Overweight	28.4	53.1	4.9	13.6	
Obesity	45.3	41.5	1.9	11.3	
LTPA					
Yes	18.8	63.6	6.7	10.9	0.073
No	23.8	54.6	3.8	17.7	
*Women (n = 630)*	17.5	63.0	4.1	15.4	
Age (years)					
18–35	14.0	64.6	5.6	15.9	0.009
36–55	22.6	60.7	2.0	14.7	
Origin					
Balearic Islands	16.4	64.2	3.9	15.6	0.186
Regions of Spain	19.8	64.9	2.3	13.0	
South America	14.9	59.7	4.5	20.9	
Other countries	30.4	47.8	13.0	8.7	
Educational level					
Low	14.6	58.3	6.3	20.8	0.373
Medium	18.3	60.5	4.2	17.0	
High	17.3	67.7	3.1	11.9	
Professional profile					
Student	12.3	59.0	4.9	23.8	0.010
Unemployed	25.0	51.8	1.8	21.4	
Employed	18.4	65.3	4.3	12.0	
BMI (kg/m^2^)					
Normal-weight	11.3	70.7	4.4	13.6	<0.001
Overweight	25.6	53.2	2.6	18.6	
Obesity	36.8	38.2	2.9	22.1	
LTPA					
Yes	18.9	66.4	2.5	12.3	0.014
No	16.3	59.0	5.9	18.9	

*Abbreviations: BMI* body mass index, *LTPA* leisure-time physical activity

^a^Values are expressed as percentages

^b^Significant trends between Weight Estimation groups have been evaluated by *χ* ^2^

^c^Significant trends between males and females have been evaluated by *χ* ^2^: †*p* < 0.05; ‡*p* < 0.01; §*p* < 0.001

### Body image satisfaction

Three quarters of the population reported body image dissatisfaction (Table 3) with significant differences between males and females (67.1% males; 81.0% females). Females were 2.84 times more likely (95%CI: 2.17–3.72) to be dissatisfied with being overweight and 0.27 times less (95%CI: 0.16–0.44) dissatisfied with being underweight than males (data not shown). The prevalence of middle-aged adults dissatisfied with their body image was higher than in the youngers; dissatisfaction with being overweight was higher in middle-aged adults, and with being underweight in young adults. Place of birth, professional profile, and employment status influenced body satisfaction. More overweight males were satisfied with their body image than females, but more overweight males were satisfied with their weight than obese males. Active participants were more satisfied with body image than no actives.

**Table 3:** Body Image Satisfaction according to socio-demographic variables, BMI distribution and LTPA^a,b,c^

	Dissatisfied being underweight	Satisfied	Dissatisfied being overweight	*P*
*Men (n = 451)*	15.3§	32.9§	51.8§	
Age (years)				
18–35	19.6§	35.8§	44.5§	<0.001
36–55	4.1	25.2†	70.7‡	
Origin				
Balearic Islands	15.8§	27.9†	56.4§	0.132
Regions of Spain	11.4†	42.0‡	46.6§	
South America	14.8	40.7‡	44.4‡	
Other countries	17.6	47.1‡	35.3‡	
Educational level				
Low	10.3	37.9†	51.7‡	0.255
Medium	18.1§	33.5§	48.4§	
High	11.7‡	30.7†	57.7§	
Professional profile				
Student	25.4‡	28.8†	45.8§	0.008
Unemployed	4.0	40.0‡	56.0‡	
Employed	12.3§	33.8§	53.9§	
BMI (kg/m^2^)				
Normal-weight	27.2§	46.5§	26.3§	<0.001
Overweight	1.9	21.1§	77.0§	
Obesity	2.0	7.8	90.2†	
LTPA				
Yes	15.3§	36.0§	48.7§	0.032
No	14.1‡	24.2†	61.7§	
*Women (n = 630)*	3.9§	19.0§	77.1§	
Age (years)				
18–35	5.6§	21.4§	73.0§	0.002
36–55	1.2	15.4†	83.3‡	
Origin				
Balearic Islands	2.9§	19.5†	77.6§	0.034
Regions of Spain	3.1†	23.3‡	73.6§	
South America	10.6	12.1‡	77.3‡	
Other countries	4.3	8.7‡	87.0‡	
Educational level				
Low	4.3	14.9†	80.9‡	0.849
Medium	4.2§	18.3§	77.5§	
High	3.5‡	20.9†	75.6§	
Professional profile				
Student	9.1‡	17.4†	73.6§	0.007
Unemployed	1.8	10.9‡	87.3‡	
Employed	2.8§	20.7§	76.6§	
BMI (kg/m^2^)				
Normal-weight	4.5§	27.5§	68.1§	<0.001
Overweight	0.6	1.9§	97.4§	
Obesity	0.0	1.5	98.5†	
LTPA				
Yes	1.9§	22.6§	75.5§	0.004
No	6.0‡	15.3†	78.7§	

*Abbreviations: BMI* body mass index, *LTPA*, leisure-time physical activity

^a^Values are expressed as percentages

^b^Significant trends between Weight Estimation groups have been evaluated by *χ* ^2^

^c^Significant trends between males and females have been evaluated by *χ* ^2^: †*p* < 0.05; ‡*p* < 0.01; §*p* < 0.001

### Weight concern

Around 50% of the study population reported that they were not worried about their weight (Table 4). More females than males reported to be worried about their weight. In both sexes, more middle-aged adults were worried about weight gain than young adults. Latin American (the largest foreign colony) and unemployed females were the most worried about weight gain. Around 45% of males and 20.7% of overweight females reported to be not worried about weight gain, and 59.2% of obese males and 24.2% of obese females also reported it. Only one in ten obese males and one in three obese females reported to be very worried about gain weight.

**Table 4:** Weight Concern according to socio-demographic variables, BMI distribution and LTPA^a,b,c^

	Not at all	A little	A lot	*P*
*Men (n = 451)*	60.6§	35.8§	3.6§	
Age (years)				
18–35	65.1§	32.9†	2.0§	0.001
36–55	48.7§	43.4†	8.0†	
Origin				
Balearic Islands	59.8§	37.0‡	3.3§	0.181
Regions of Spain	57.3‡	39.0	3.7†	
South America	58.3‡	29.2	12.5†	
Other countries	81.3‡	18.8	0.0	
Educational level				
Low	57.1†	32.1	10.7	0.165
Medium	63.8§	33.2‡	3.0§	
High	56.1§	40.5†	3.4§	
Professional profile				
Student	62.3§	34.2	3.5§	0.935
Unemployed	52.2†	43.5	4.3†	
Employed	60.1§	36.2§	3.7§	
BMI (kg/m^2^)				
Normal-weight	71.5§	27.1§	1.4§	<0.001
Overweight	45.1§	50.7	4.2§	
Obesity	59.2§	28.6	12.2†	
LTPA				
Yes	59.2§	37.0‡	3.8§	0.864
No	62.1§	34.5†	3.4§	
*Women (n = 630)*	34.1§	47.8§	18.1§	
Age (years)				
18–35	38.5§	42.2†	19.4§	0.003
36–55	27.4§	56.5†	16.1†	
Origin				
Balearic Islands	34.2§	49.9‡	16.0§	0.019
Regions of Spain	37.2‡	47.9	14.9†	
South America	27.4‡	37.1	35.5†	
Other countries	38.1‡	42.9	19.0	
Educational level				
Low	28.3†	54.3	17.4	0.512
Medium	36.1§	44.4‡	19.4§	
High	32.6§	51.0†	16.3§	
Professional profile				
Student	37.4§	40.9	21.7§	0.052
Unemployed	26.5†	42.9	30.6†	
Employed	33.8§	50.4§	15.8§	
BMI (kg/m^2^)				
Normal-weight	39.3§	48.5§	12.3§	<0.001
Overweight	20.7§	51.0	28.3§	
Obesity	24.2§	46.8	29.0†	
LTPA				
Yes	35.6§	49.1‡	15.2§	0.169
No	32.4§	46.3†	21.3§	

*Abbreviations: BMI* body mass index, *LTPA* leisure-time physical activity

^a^Values are expressed as percentages

^b^Significant trends between Weight Estimation groups have been evaluated by *χ* ^2^

^c^Significant trends between males and females have been evaluated by *χ* ^2^: †*p* < 0.05; ‡*p* < 0.01; §*p* < 0.001

### Current dieting

Fourteen per cent of the study population (18.2% of overweight and 30.0% of obese) reported current dieting (Table 5). More active males reported current dieting (14.5%) than inactive males (7.3%).

**Table 5:** Current Dieting according to socio-demographic variables, BMI distribution and LTPA^a,b,c^

	Yes	No	*P*
*Men (n = 451)*	14.0	86.0	
Age (years)			
18–35	10.7	89.3	0.107
36–55	16.3	83.7	
Origin			
Balearic Islands	13.0	87.0	0.460
Regions of Spain	13.6	86.4	
South America	12.5	87.5	
Other countries	0.0	100.0	
Educational level			
Low	10.7	89.3	0.964
Medium	12.5	87.5	
High	12.3	87.7	
Professional profile			
Student	12.0	88.0	0.972
Unemployed	11.5	88.5	
Employed	12.7	87.3	
BMI (kg/m^2^)			
Normal-weight	7.1	92.9	0.001
Overweight	15.6	84.4	
Obesity	24.5	75.5	
LTPA			
Yes	14.5	85.5	0.038
No	7.3	92.7	
*Women (n = 630)*	15.3	84.7	
Age (years)			
18–35	14.2	85.8	0.316
36–55	17.1	82.9	
Origin			
Balearic Islands	15.9	84.1	0.368
Regions of Spain	10.9	89.1	
South America	19.7	80.3	
Other countries	13.0	87.0	
Educational level			
Low	16.7	83.3	0.782
Medium	16.0	84.0	
High	14.1	85.9	
Professional profile			
Student	14.2	85.8	0.616
Unemployed	19.6	80.4	
Employed	15.0	85.0	
BMI (kg/m^2^)			
Normal-weight	10.5	89.5	<0.001
Overweight	20.9	79.1	
Obesity	34.3	65.7	
LTPA			
Yes	17.2	82.8	0.227
No	13.7	86.3	

*Abbreviations: BMI* body mass index, *LTPA* leisure-time physical activity

^a^Values are expressed as percentages

^b^Significant trends between Weight Estimation groups have been evaluated by *χ* ^2^

^c^Significant trends between males and females have been evaluated by *χ* ^2^: †*p* < 0.05; ‡*p* < 0.01; §*p* < 0.001

## Discussion

Self-perception of body shape is an important factor in weight control practices [28–34]. This study has assessed body weight satisfaction, acceptance of body image, weight concerns and dieting habits among a Mediterranean adult population. Main findings of the study were that three quarters of the studied Mediterranean adult population reported body image dissatisfaction, males with underweight and females with overweight; middle-aged adults perceived their body shape to be larger and were more likely to underestimate their body weight; half of the population were not worried about their body weight, but females were more worried than males; employment status, origin of people, BMI status, and to be active or not are factors that influence body satisfaction.

Self-perceived body image is an important factor for eating habits and life-style behaviors. While some researchers proposed body weight dissatisfaction as an important factor in preventing weight gain and promoting weight loss [28, 29], other researchers proposed body weight dissatisfaction as a driver of unhealthy dieting behaviors [30, 31]. Despite males showing higher excessive weight prevalence, females were more likely to be dissatisfied with their body image than males, reflecting that body and physical appearance seem to be more important in females than males, which are in align with previous studies [32, 33]. As it has been above mentioned, the ‘ideal body’ usually managed by popular culture and mass media is a tall and thin frame which typically does not reflect average women developing severe opinion of their self-image and hence unnecessarily worried about their weight [32–34]. Of importance, our results support notion that females are more likely to be concerned with their body weight compared to males [34].

Our results also support the results of previous studies that showed that older participants were more likely to underestimate their current weight [10], and to be dissatisfied with their body image. Young people tend to perceive themselves as having higher body weight than older people do [21, 35–37], and in accordance with previous studies, the desire to lose weight increases with the age in males [38].

Some studies showed that the unemployed population perceived themselves as having bad health [39, 40]. Unemployment is also linked to increased health factor risks such as obesity [41], amongst other habits such as smoking and alcohol and drug consumption. Our results are in accordance with a previous work [42], which showed that unemployed women were more concerned with their appearance.

Reported body image differences among females from different countries and ethnicities have also been reported previously [20], suggesting the need to include nationality and place of birth in the evaluation of body image [43]. Our results showed that Latin American females were more likely to be dissatisfied with being underweight and were more worried about this than their counterparts. Controversial results were obtained in previous studies of body image perception among Latin American females [28, 44]. Despite the fact that Latin American females showed a higher preference for a voluminous body shape than women born in the USA [28], other study [44] reported that Latin America females showed the same body image dissatisfaction as females born in USA. In fact, in some cultures larger body size was traditionally linked to prosperity [10]. Previously, it has been also reported similar body image dissatisfaction among Latin American, Asian or Caucasian females [45]. On the other hand, in 2002, a Spanish study revealed that Mexican females were more likely to be dissatisfied with their bodies than Spanish females, showing a higher preference for slimmer bodies [46].

Despite media mass and health policies being focused on raising awareness about healthy weights, an increasing number of participants with excessive weight failed to recognize that their weight is a cause of concern [47]. While the general image that the media mass uses is based on slimmer bodies, the severe images that are used to define obesity should be linked to the impression that extremely high weight images are required to define excessive weight [47, 48]. Fortunately along these lines, our results reported that our overweight participants were more concerned about their weight than normal-weight participants were.

Some studies investigated the relationship between LTPA and body image satisfaction with a generally repeated conclusion: LTPA is related to body image satisfaction [48]. Participants doing LTPA are more likely to be satisfied with their body image than participants not doing LTPA with the same BMI [49].

Despite the results showing that only approximately 25% of the population reported body image satisfaction, just 14.0% of the population was dieting. Our results, in concordance with earlier studies [50, 51], showed that heavier participants were more likely to be on a diet. Moreover, our results highlight that males doing LTPA were more likely to be on diet, which is in agreement with a previous study that reported that masculine gender role endorsement appears to be associated with the development of muscularity-related eating and body image concerns [52].

Finally, the relationship between ideal body image and weight self-regulation process may be found in Higgins’ [53, 54] regulatory focus theory (RFT). According to RFT, the personal motivation to lose or to maintain weight may be triggered to show an attractive body or to avoid social refection of fatness, being last case more specifically influential on individual who perceives himself/herself more deviant from socially body acceptable. Moreover, for the individual, losing weight is faced by obstacles on psychological, cognitive and emotional levels due to sacrifices demanded by weight self-regulatory behaviors that may dissuade many males and females. Moreover, an “ideal state” of prescription and enforcement of body shape looks like is a very sensitive, political and controversial topic that should be arranged someday. Nevertheless, new education and/or awareness strategies for promoting positive body image attitudes are needed, as well as it is compulsory to define the role that different healthcare providers, or community members, or other professions can play in promotion positive self-esteem and body image.

## Conclusions

Three quarters of the studied Mediterranean adult population reported body image dissatisfaction, but almost half of the population reported that they were not worried about their body weight. In particular, females were more likely to be concerned about their body weight status compared with males, and practice of physical activity is a positive factor in self-perception. Holistic strategies are needed to avoid promoting unreal bodies. New strategies that include education, awareness, health, social education, etc., should be developed. In the next future, a new social environment which fosters the perception and acceptance of the real body image, avoiding distortions, and therefore future pathologies, should be developed.

## Abbreviations

BMI: Body mass index
CI: Confidence interval
LTPA: Leisure-time physical activity
OR: Odds ratio
SPSS: Statistical Package for the Social Sciences
WHO: World Health Organization
